# 

**Published:** 1977

**Authors:** 


					jM'?-
. ? ?>f . ^3 C ? ?'' -,'V* ? .- ???'*pWSffilPI- - . it->.????'M
4
Ml^l
?? < ?:
BPsSll
?M, ?,''??' feiw--'.3
giij
inm?
I Mi
?f"r
Wmm? I
VOllE^I
'r!?
U-'Ji -?r* -;'- " '<>- ?*& '-? V^-v.v^ ?sf -v^4a
KgKt.\*$Sra^|?& . /jQjg
,v i'-
; .;., ,-:'i'-iS!'":"-?"
., * II ' fc 's
* ..N''': ;V~..*
.&.t?&&.,r.i, ..^ ?, .. . a.*   _.l.,,.^.^ '
jANUARY/APRIL, 1977
Vol. 92 (i/ii) No. 341/342

				

